# Proto-Oncogenes and Cell Cycle Gene Expression in Normal and Neoplastic Oral Epithelial Cells Stimulated With Soluble Factors From Single and Dual Biofilms of *Candida albicans* and *Staphylococcus aureus*

**DOI:** 10.3389/fcimb.2021.627043

**Published:** 2021-02-25

**Authors:** María Isabel Amaya Arbeláez, Ana Carolina Alves de Paula e Silva, Geovana Navegante, Valeria Valente, Paula Aboud Barbugli, Carlos Eduardo Vergani

**Affiliations:** ^1^Laboratory of Applied Microbiology, Faculty of Dentistry, Department of Dental Materials and Prosthodontics, São Paulo State University (Unesp), Araraquara, Brazil; ^2^Laboratory of Molecular and Cell Biology, School of Pharmaceutical Sciences, Department of Clinical Analysis, São Paulo State University (Unesp), Araraquara, Brazil

**Keywords:** biofilms, *Candida albicans*, *Staphylococcus aureus*, metabolites, oral cancer, gene expression

## Abstract

This study was aimed at analyzing proto-oncogenic signaling pathway activation in normal oral keratinocytes (NOK-si) and neoplastic cell lines (SCC 25 and Detroit 562) stimulated with metabolites (soluble factors) from single and dual biofilms of *Candida albicans* and *Staphylococcus aureus*. Soluble factors (SF) from early (16-h) and mature (36-h) biofilms of *C. albicans* and *S. aureus* were collected and incubated with cell cultures, which were subsequently evaluated using gene expression via RT-qPCR, cell viability via AlamarBlue^TM^, and flow cytometry cell cycle analysis. In general, exposure to the SF of early and mature biofilms from *C. albicans* and dual species caused a major reduction in NOK-si cell viability and enhanced the sub G0 phase. This led to a decrease in gene expression. However, in this cell line, SF of *S. aureus* biofilms upregulated the *CDKN1A* gene followed by the maintenance of cell viability and a significant increase in the G2/M population. For tumor cells, SCC 25 and Detroit 562, the stimuli of SF biofilms upregulated oncogenes such as *hRAS* and *mTOR*, as well as *Bcl-2* and *CDKN1A*. SCC 25 and Detroit 562 cells could survive even after 24 h of stimuli from both SF (early and mature). This occurred without significant changes taking place in the cell cycle progression for SCC 25, and with a significant tendency to increase the G2/M phase for Detroit 562. These results point to the fact that metabolites from prevalent clinical fungal and bacterial biofilms, *C. albicans* and *S. aureus*, can disrupt the homeostasis of normal and neoplastic oral epithelial cells. This changes proto-oncogenes’ expression, specifically *PI3KCA*, *hRAS*, *mTOR*, *BRAF*, and cell cycle genes *CDKN1A* and *Bcl-2*, thus causing a disturbance in cell viability, survival, and the cell cycle profile.

## Introduction

Cancer is a disease characterized by uncontrolled cell division caused by unbalances between proliferation and cell death processes. Tumor development and progression are associated with several changes in the activity of cell cycle regulators. Thus, failures in cell cycle inhibitors and excessive signaling of positive regulators of cell division can lead to cancer. In most cases, the changes accumulated during tumor progression confers the initial competencies that allow uncontrolled cell division, such as sustained proliferation signaling, escape from growth suppression and cell death, and replicative immortality (Hanahan and Weinberg, 2011). Once uncontrolled cell proliferation is established, other genetic alterations are favored to the tumor progression takes place.

In this study, we focused on head and neck squamous cell carcinoma (HNSCC). Around 890,000 new cases of (HNSCC) (including the oral cavity, pharynx, and larynx) and 450,000 deaths related to HNSCC were estimated to occur worldwide in 2018 (Bray et al., 2018). Oral cavity squamous cell carcinoma (OSCC) made up most of these cases, leading to changes in individual's functional and aesthetic aspects, and decreasing their quality of life and a five-year mortality rate of nearly 50% (International Agency for Research on Cancer, 2012). HNSCC development is a multistep process (Pai and Westra, 2009). The accumulation of several genetic alterations in oncogenes and tumor suppressor genes results in the destabilization of cellular growth control systems (Derka et al., 2006). Some pathways implicated in the molecular settings of OSCC progression are p53 protein, transforming growth factor beta 1 (TGF1β), epidermal growth factor receptor (EGFR) and cyclins, anaplastic lymphoma kinase, wingless homeobox genes (WNT), and mammalian target of rapamycin (*mTOR*). (Kumar et al., 2017) The EGFR/RAS/RAF/MEK/ERK and EGFR/PI3K/AKT/*mTOR* signaling play a pivotal role in many aspects of these cellular events, including proliferation, differentiation, and survival (Carracedo et. al., 2008; Peng et al., 2018). For instance, the most significant risk factors for HNSCC include tobacco use, alcohol consumption, and human papillomavirus infections (HPV) (Alfouzan, 2019).

The development of HNSCC not associated with significant risk factors, as HPV infections, is most closely associated with a poor prognosis, and the reason is still unclear. Recent scientific advances have significantly contributed to understanding the connection between the microbiome and cancer. The human body continuously is exposed to microbial cells resident and transient, as well as their byproducts, including toxic metabolites. Circulation of toxic metabolites may contribute to cancer onset or progression at locations distant from where a particular microbe resides (Rajagopala et al., 2017). The oral microbiota has been associated with development by several mechanisms of action. Bacteria could provoke an inflammatory response. The inflammatory mediators like interleukins, tumor necrosis factor-alpha (TNF-α), and the matrix metalloproteinases MMP8 and MMP9 produced, facilitate cell proliferation, mutagenesis, and oncogene activation. Another mechanism is related to the secretion of bacterial effector proteins using type III or type IV secretion systems (T3SS/T4SS), which could affect cell proliferation, cytoskeletal rearrangements, activation of NF-κβ, and inhibition of cellular apoptosis. Finally, bacteria can produce carcinogenic substances (Zhang et al., 2018).

The oral microbiome is a diverse community of microorganisms in the human oral cavity (Perera et al., 2016). Approximately 700 resident species, including viruses, archaea, protozoa, bacteria, and fungi, are involved in a wide variety of functions that are important for maintaining and restoring one's oral health (Kilian et al., 2016). However, their role in the etiology and predisposition of HNSCC has not been thoroughly characterized (Karpinski, 2019). Oral microorganisms can develop biofilms on both biotic and abiotic surfaces. One of the consequences of this development is the production of some molecules (metabolites), which could change the host immune response and lead to host cell protein degradation, chronic inflammation, cellular apoptosis inhibition, gene expression modification, and the inappropriate activation of cell proliferation (Palavecino, 2004; Perera et al., 2016; Rajagopala et al., 2017; Karpinski, 2019; Zhang et al., 2020).

The relation between bacteria and cancer gained relevance when was demonstrated the pathogenic role of *Helicobacter pylori* in gastric cancer (Marwick, 1990). Subsequent studies associate carcinogenicity with bacteria in other organs. Take, for example, the increased risk of gallbladder carcinoma when associated with *Salmonella thypi* infection (Scanu et al., 2015); the relationship between lung cancer and *Chlamydia pneumonia* infection (Chaturvedi et al., 2010); colon carcinoma and *Streptococcus bovis* (Ellmerich et al., 2000); or pancreatic cancer and *Malassezia* (Aykut et al., 2019). The periodontal pathogens *Porphyromonas gingivalis* and *Fusobacterium nucleatum* were found to be able to stimulate tumorigenesis in an oral epithelial cell murine model through toll-like receptors (Binder Gallimidi et al., 2015). On the other hand, the role of some fungi, such as *C. albicans*, has also been deemed an etiologic factor for potentially malignant disorders (Bakri et.al., 2010; Sankari et al., 2015). The review of Ramirez-Garcia highlighted that this species has the ability to produce carcinogen metabolites that are capable of inducing functional and structural alterations in deoxyribonucleic acid (DNA) replication and protein function, promoting disturbances in the cell cycle, and allowing the activation of oncogenes (Ramirez-Garcia et al., 2016). Zhang et al. described the relationship between the oral mucosal microbial profile and OSCC in clinical samples. Species included *Fusobacterium nucleatum*, *Prevotella intermedia*, *Aggregatibacter segnis*, *Peptostreptococcus stomatis*, and *Catonella morbi*, which reside in the oral mucosa as commensals, could be opportunistic pathogens with potential correlations with OSCC. *Fusobacterium*, *Alloprevotella* and *Porphyromonas*, were prevalent in cancer tissues, whereas *Streptococcus*, *Veillonella*, and *Rothia*, significantly decreased (Zhang et al., 2020). Studies have revealed that oral infections involving *Candida albicans*, and *Staphylococcus aureus*, are related to the development of head and neck oncogenesis or perhaps at some stages of tumor progression (Chocolatewala et al., 2010; Arzmi et al., 2019). The prevalence of *C. albicans* and *S. aureus* in the oral cavity has already been described (Ribeiro et al., 2012), mainly at mucous membrane sites and acrylic denture resins. *C. albicans* was isolated in 66.7% of the dentures, whereas *S. aureus* in 49.5%. *C. albicans* was isolated in 86% of the patients with atrophic denture stomatitis, and S. aureus in a similar percentage (84% of patients) (Baena-Monroy et al., 2005).

In a prior study by our group, *C. albicans* and *S. aureus* biofilms produced high amounts of proteinase and phospholipase-C (Zago et al., 2015). In another study, were observed that metabolites from the mature biofilms of *C. albicans* and *S. aureus* (36-h), denominated soluble factors (SFs) promoted inflammatory response and oral keratinocytes death. Moreover, the SFs from dual-species biofilms were the most toxic to the cells (de Carvalho Dias et al., 2017). Furthermore, *S. aureus* α-toxins can induce cytotoxicity and inflammation mediated by TNF-α in keratinocytes, upregulation of pro-inflammatory genes, and suppression of MAPK signaling pathway (Kirker et.al., 2009; Secor et al., 2011). Based on these results and on literature reports, this study aimed to evaluate whether these SFs, from early (16-h) and mature (36-h) biofilms, would be able to activate gene expression related to oncogenic signaling pathways and tumor progression. For this, normal and neoplastic oral epithelial cells were challenged with short (4 h) and long (24 h) stimuli with sterile SF containing metabolites from 16-h and 36-h of single and dual-species biofilms of *C. albicans* and *S. aureus*. The effect of each SF on *CDKN1A, CDKN1B*, *PI3KCA*, *Bcl-2*, *STAT3*, *TP53*, *MEK1*, *AKT*, *mTOR*, *hHAS*, *RB1*, *and BRAF* gene expression was evaluated, along with cell viability and the cell cycle profile.

## Materials and Methods

### Candida albicans and Staphylococcus aureus Growth Conditions

*C. albicans* SC5314 (ATCC MYA-2876) and *S. aureus* ATCC 25923 were obtained from the American Type Culture Collection. *C. albicans* and *S. aureus* were subcultured from frozen stocks on Sabouraud Dextrose Agar plates (SDA, Acumedia® Manufacturers Inc., Baltimore, MD, USA) supplemented with chloramphenicol (0.1 g/L), as well as brain heart infusion agar plates (BHI- Acumedia® Manufacturers Inc., Baltimore, MD, USA) supplemented with amphotericin B (0.025 g/L), respectively. For pre-inoculum, 10 freshly grown colonies of *C. albicans* were transferred to Yeast Nitrogen Base broth culture medium (YNB- Difco^TM^, Becton Dickinson Sparks, MD, USA) supplemented with 100 mM of glucose. For *S. aureus*, seven colonies were transferred to Tryptic Soy Broth medium (TSB, Acumedia®, Manufacturers Inc., Baltimore, MD, USA). Both were grown at 37^o^C for 18 h. Each pre-inoculum was diluted 1:20 in its respective medium and was incubated at 37^o^C until the Midlog phase 4 h for *S. aureus* (10^8^ CFU/ml), and 8 h for *C. albicans* (10^6^ CFU/ml). The final Midlog times were synchronized. Afterward, the inoculum was centrifuged (4,000 rpm, 4°C, 10 min), and the pellet was harvested and washed twice with sterile phosphate-buffered saline solution (PBS, pH 7.2). Microorganisms were resuspended in RPMI-1640 culture medium (Sigma-Aldrich®, St Louis, MO, USA) supplemented with HEPES (25 mM), L-glutamine (2.0 mM), and sodium bicarbonate (2.0 g/L) (Sigma-Aldrich®, St Louis, MO, USA) (Dias et al., 2016). The optical densities were standardized to 1 × 10^7^ CFU/ml for both microorganisms.

### Biofilm Formation and Metabolite Recovering

The literature reports *C. albicans* SC5314 and *S. aureus* 25923 strains like thick biofilm formers. Scanning Electron Microscopy, Crystal Violet (biomass), and the virulence factors production described by Zago et al. (2015), besides the proteomic assays described by Peters et al. (2010), confirm these biofilm characteristics. Routinely and as experimental quality controls of these biofilms, Nile Red and Syto 9 staining, in confocal analysis, presented thickness values around 50 to 100 μm (data not shown). The biofilm formation protocol was realized according to Zago et al. (2015), Dias et al. (2016), and de Carvalho Dias et al. (2017). Briefly, single- and dual-species biofilms were carried out in 24-well microplates (TPP Techno Plastic Products AG, Switzerland). Then, 750 µl of RPMI-1640 and 750 µl of inoculum were added separately in each well. In the case of dual-species biofilms, 750 µl of each inoculum were added per well. The plates were incubated for 90 min (adhesion phase) at 75 rpm at 37°C. Afterward, the medium was removed, and the wells were washed twice with PBS 1X to remove the non-adhered cells. Then, 1.5 ml of fresh RPMI-1640 was added to each well. The plates were placed on an orbital shaker at 37°C for 16-h (early) and 36-h (mature) to obtain the biofilms (Zago et al., 2015; Dias et al., 2016; de Carvalho Dias et al., 2017). To obtain the metabolites (SF), the biofilms were carefully scraped with the medium, using a sterile plastic tip for 1 min (Silva et al., 2010). The suspension was homogenized, filtered with a low-protein binding filter (SFCA 0.22 μm, Corning®, NY, USA), and stored at −20^o^C prior to its use.

### Cell Culture Conditions

The cell lines of NOK-si, SCC 25, and Detroit 562 were cultured in 75-cm^2^ flasks maintained in a humidified atmosphere at 37°C and 5% CO_2_. At 90% confluence, cells were washed with PBS 1X, detached with trypsin solution (0.05%)/EDTA (0.53 mmol. L-1) (Sigma-Aldrich®, St Louis, MO, USA), and centrifuged at 400 × *g* for 5 min. The cells were suspended in a fresh cell culture medium, stained with trypan blue (ratio 1:1), and counted using the Countess II FL (Life Technologies, Carlsbad, CA, USA). The NOK-si (normal oral keratinocyte spontaneously immortalized) (Castilho et al., 2010) came from Professor Dr. Carlos Rossa, Jr. from the Cellular and Molecular Biology Laboratory, Department of Periodontics, School of Dentistry, São Paulo State University (UNESP). It was maintained in Dulbecco’s Modified Eagle’s Medium with 4.5 g/L of glucose (DMEM- Lonza^TM^ BioWhittaker^TM^, BS, Switzerland), 2.0 mM of L-glutamine (Sigma-Aldrich®, St Louis, MO, USA), and 10% fetal bovine serum (FBS- Gibco^TM^, Thermo Fischer Scientific Inc., MA, USA). The SCC 25 (tongue squamous cell carcinoma cell line) was purchased from the Rio de Janeiro Cell Bank (BCRJ- code 0194) and cultured in a 1:1 mixture of Dulbecco’s Modified Eagle’s Medium (DMEM) and Ham’s F12 Medium containing 1.2 g/L of sodium bicarbonate (Lonza^TM^ BioWhittaker^TM^, BS, Switzerland), 2.5 mM of L-glutamine, and 15 mM of HEPES, supplemented with 400 ng/ml of hydrocortisone (Sigma-Aldrich®, St Louis, MO, USA) and 10% FBS. The Detroit 562 (pharyngeal Metastatic Epithelial Cell) was purchased from BCRJ (code 0076) and cultured in DMEM with 1.0 g/L of glucose, 1% non-essential amino acids (Lonza^TM^ BioWhittaker^TM^, BS, Switzerland), 2 mM of L-glutamine, 1 mM of sodium pyruvate (Sigma-Aldrich®, St Louis, MO, USA), 1,500 mg/L of sodium bicarbonate, and 10% FBS.

### Cell Viability Assay

Cells (2.0 × 10^4^ cells/well) were plated into 96-well black polystyrene plates (Corning®, NY, USA) maintained in a humidified atmosphere at 37°C and 5% CO_2_. Cells were stimulated for 4 h and 24h with SF from 16-h and 36-h of single and dual-species biofilms. Following the stimulation, AlamarBlue^TM^ reagent (Invitrogen^TM^, Thermo Fischer Scientific Inc., MA, USA) was added (20 μl/well) and incubated for 4 h at 37°C. For the control of living cells, the cells were grown under standard conditions in RPMI-1640 medium (processed in the same way as the analyzed samples). For the cell death control, the cells were treated with Triton^TM^ X-100 (Sigma-Aldrich®, St Louis, MO, USA). Fluorescence measurements were performed on Fluoroskan Ascent® FL (Thermo Fisher Scientific Inc., MA, USA) equipment with 544-nm excitation filters and 590-nm emission filters. The assay was performed in triplicate on at least three separate occasions. The fluorescence measurements were converted to percentages based on the control without a stimulus. Non-normal values were analyzed using the Shapiro-Wilks test. In addition, the Kruskal-Wallis test was applied along with the Dunn's Multiple Comparison Test using GraphPad PRISM vs 8.0 software.

### Cell Cycle Assay

NOK-si, SCC 25, and Detroit 562 cells (2.5 × 10^5^ cells/well) were seeded into six-well plates (Corning®, NY, USA), maintained in a humidified incubator at 37°C and 5% CO_2_. Cells were stimulated for 4 h and 24 h with SFs from 16-h and 36-h of single and dual-species biofilms. For the control, cells were grown under standard conditions in RPMI-1640 medium (processed in the same way as the analyzed samples). The monolayer was dissociated and detached with 200 μl of Accutase solution (SIGMA) for 10 min at 37^o^C. Afterward, 800 μl of PBS were added, and the suspensions were carefully homogenized and transferred to microtubes. The microtubes were centrifuged at 400 × *g* for 5 min at 4^o^C, and finally, the supernatant was discarded through inversion. The pellet was carefully resuspended in 100 μl of 1× PBS, and 900 μl of chilled 70% ethanol was gently added to the microtube wall while it was being slowly vortexed. Microtubes were incubated overnight at 4^o^C. After this period, the suspension was centrifuged at 700 × *g* for 5 min at 4^o^C. The supernatant was discarded through inversion, and the pellet was carefully resuspended in 100 μl of 1× PBS. After this came the addition of 100 μl of a cell cycle solution (3.4 mM of tris HCl, pH 7.4; 0.1% Triton X-100; 700 U/L of RNAse (DNAse free); 10 mM of NaCl (Sigma-Aldrich®, St Louis, MO, USA); and 30 μg/ml of propidium iodide (Invitrogen^TM^, Thermo Fischer Scientific Inc., MA, USA). The tubes were incubated for at least 20 min on ice protected from light. The suspension was transferred to a cytometry tube and subjected to analysis via flow cytometry (BD FACSAria ™ III cell sorter). With BD FACS Diva software, a side-scatter (SSC) versus forward-scatter (FSC) dotplot graphic was built to locate the homogeneous cell population. This population was derived for an SSC-A x SSC-W dotplot graphic, followed by an FSC-A x FSC-W graphic, to exclude debris and doblets. Following this population, dotplot graphic PE-A (filter for the fluorescence of the propidium iodide marker) x PE-W was made to determine the labeled cells. From this population were acquired 10,000 events in low-acquisition mode. The distribution of the cell cycle phases was followed by a histogram graph with PE-A for the X-axis on a linear scale. The distribution of the cell cycle phases was analyzed using the Watson Pragmatic model by the FlowJo ™ Software (Windows) vs 10 program.

### RNA Extraction and Quantitative RT-PCR

NOK-si, SCC 25, and Detroit 562 cells (2.5 × 10^5^ cells) were seeded into 25-cm^2^ cell culture flasks with filters (Corning®, cat. 430639) and stimulated for 4 h (37°C, 5% CO_2_) with SF from 16-h and 36-h of single and dual-species biofilms. For the control, the cells were grown under standard conditions in RPMI-1640 medium (processed in the same way as the analyzed samples). The total ribonucleic acid (RNA) was isolated from 1 × 10^6^ cultured cells using Tizol® Reagent (Invitrogen^TM^, Thermo Fischer Scientific Inc., MA, USA) according to the manufacturer’s protocol. The concentration and purity of the samples were assessed via absorbance using NanoDrop^TM^ 2000 (Thermo Fisher Scientific Inc., MA, USA). The mean ratio value of A_260_/A_280_ for all RNA samples was 1.81 (± 0.06), reflecting high purity. The RNA was reverse transcribed into cDNA using the High Capacity cDNA Reverse Transcription kit (Applied Biosystems®, CA, USA) according to the fabricant’s recommendations. This occurred after the treatment of RNA with DNAse I (Invitrogen^TM^, Thermo Fischer Scientific Inc., MA, USA) in the presence of an RNAse inhibitor (RNAseOUT- Invitrogen^TM^, Thermo Fischer Scientific Inc., MA, USA). The relative mRNA expression was quantified using real-time PCR analysis in the Gene Amp® 7500 Sequence Detection System (Applied Biosystems®, CA, USA). Amplification products were detected with SYBR Green PCR Master Mix (Applied Biosystems®, CA, USA). *HPRT* was used as an internal control (Valente et al., 2009). The assay was performed in triplicate on at least two independent assays. The relative expression level was calculated by adopting the 2^-ΔΔCt^ method (Livak and Schmittgen, 2001). The fold change values were statistically analyzed using confidence intervals (CI), with a confidence level of 95% used in GraphPad PRISM software. The Primers (F: forward and R: reverse) were designed according to the parameters Tm (melting temperature): 62–64°C, GC%: 55%–60%, 3 'tail GC%: 40%, maximum size of the amplified sequence: 90–150 nucleotides using the OligoExplorer 1.2 software. The sequences were synthesized by Integrated DNA technologies, Inc. (IDT-Coralville, IA, USA). The Primers were diluted according to the manufacturer's recommendations and quantified by NanoDropTM 2000/2000c spectrophotometer (ThermoFisher Scientific). The oligonucleotides sequences used are listed in **Table 1**.

**Table 1:** Oligonucleotides sequences.

Gene	Forward primer	Reverse primer
*MEK1*	CGTGGGCACAAGGTCCTACA	CTCCCAACCGCCATCTCTAC
*CDKN1B*	ACGGGGTTAGCGGAGCAATG	CCACAGAACCGGCATTTGGG
*TP53*	GCTGAATGAGGCCTTGGAAC	TTATGGCGGGAGGTAGACTG
*CDKN1A*	TGGAGACTCTCAGGGTCGAA	CTTCCTGTGGGCGGATTAGG
*RB1*	CAGAAGGTCTGCCAACACCA	GAGCACACGGTCGCTGTTAC
*HRAS*	GGCTTCCTGTGTGTGTTTGC	AGCCAGGTCACACTTGTTCC
*AKT1*	TCGGGCTCACCCAGTGACAA	TTGCCCAGCAGCTTCAGGTA
*STAT3*	ACCATTGACCTGCCGATGTC	CCGAGGTCAACTCCATGTCA
*BCL2*	CAACATCGCCCTGTGGATGA	GCCGTACAGTTCCACAAAGG
*BRAF*	AGGCGTCCTTAGCAGAGACT	AGGGCTGTGGAATTGGAATGG
*MTOR*	CCAGCTCAGATGCCAATGAG	GGAGGAGGTTCCGAAGATAG
*PI3KCA*	TAGGCAAGTCGAGGCAATGG	GGTCGCCTCATTTGCTCAAC

## Results

### Cell Viability Profile

**Figure 1:**
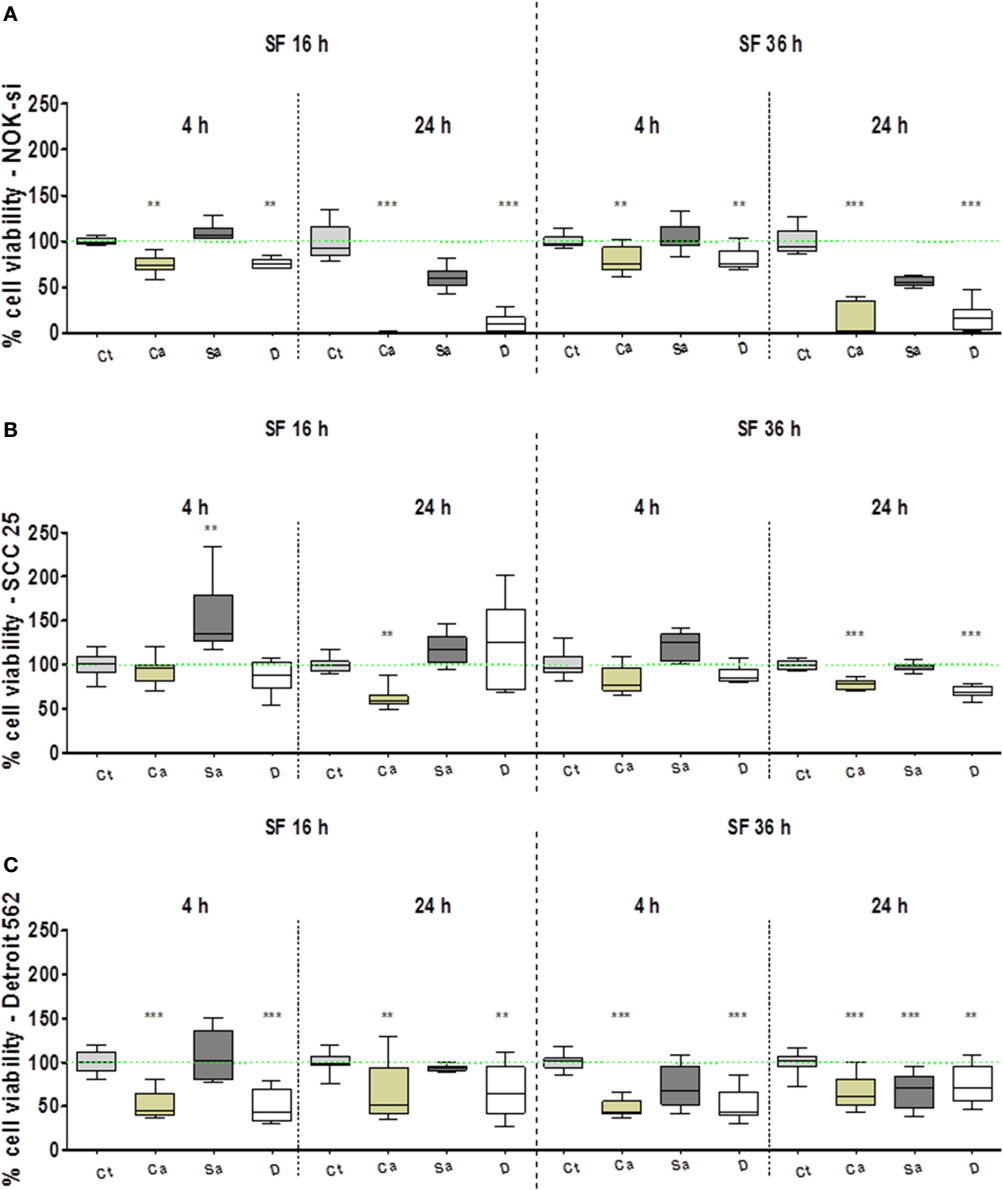
Cell viability profile. AlamarBlue^TM^ assay performed to evaluate the viability profile of cell lines NOK-si **(A)**, SCC 25 **(B)**, and Detroit 562 **(C)** upon 4 h and 24 h of stimulation with soluble factors (SF) from *C*. *albicans* (Ca), *S. aureus* (Sa), and dual species (D) of early (16-h) and mature (36-h) biofilms. One-way analysis of variance, Kruskal-Wallis test, and Dunn's Multiple Comparison Test [*compared with the control (Ct) without a stimulus]. **p < 0.01, ***p < 0.001.

The AlamarBlue^TM^ assay was performed to evaluate the viability profile of NOK-si, SCC 25, and Detroit 562 cell lines after 4 h and 24 h of stimulation with SF from *C. albicans* (Ca), *S. aureus* (Sa), and the dual species (D) of 16-h and 36-h biofilms (**Figure 1**). In general, the normal cell line of NOK-si was more susceptible after 24 h of stimulation with SF from biofilms compared with the neoplastic cell lines. It was observed that the cell viability profile of NOK-si (**Figure 1A**) after stimulation with SF from the 16-h biofilm was similar to that of the 36-h one at both times. The cell viability index after stimulation with SF from Sa showed no significant differences when compared with control Ct, whereas SF from Ca and D conditions promoted an important statistical difference, mainly after a 24-h stimulus, at which time a decrease in cell viability was observed. Similar results were also noted for Detroit 562 cells after 24 h of stimulation with SF fromthe 36-h biofilm. Apparently, SCC 25 cells after 4 hof stimulation with SF from Sa of the 16-h biofilm showed asimilar or higher viability index when compared with control Ct (**Figures 1B, C**). However, those cells showed a reduction in the viability index after 24 h of stimulation with SF from Ca. This viability reduction was also observed after 24 h of stimulation with SF from Ca and D of the 36-h biofilm. In general, metastatic cell line Detroit 562 showed an approximate 50% decrease in the cell viability index, and these cells could survive even after 24 h of stimulation.

### Cell Cycle Distribution

**Figure 2:**
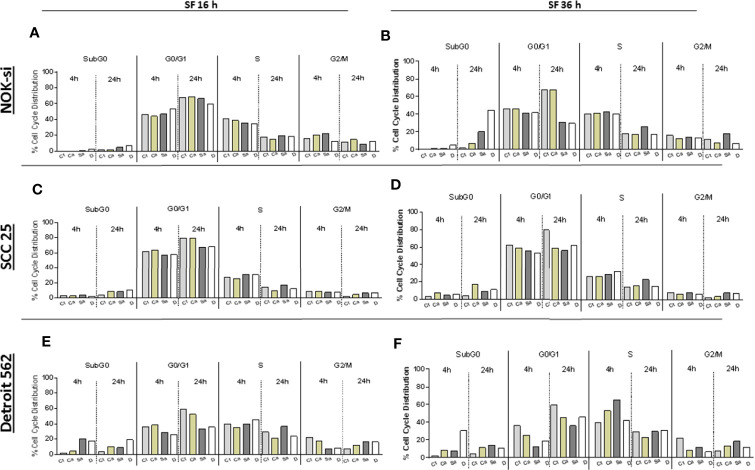
Cell cycle distribution index. NOK-si **(A)**, SCC 25 **(B)**, and Detroit 562 **(C)** cell lines stimulated with soluble factors (SF) from *C. albicans* (Ca), *S. aureus* (Sa), and dual species **(D)** from early (16-h) **(A, C, E)** and mature (36-h) **(B, D, F)** biofilms. Cells were fixed and labeled with propidium iodide, and their DNA content was measured using flow cytometry. The cell cycle distribution index (SubG0, G0/G1, S, G2/M) was analyzed after 4 and 24 h of stimulation.

**Figure 3:**
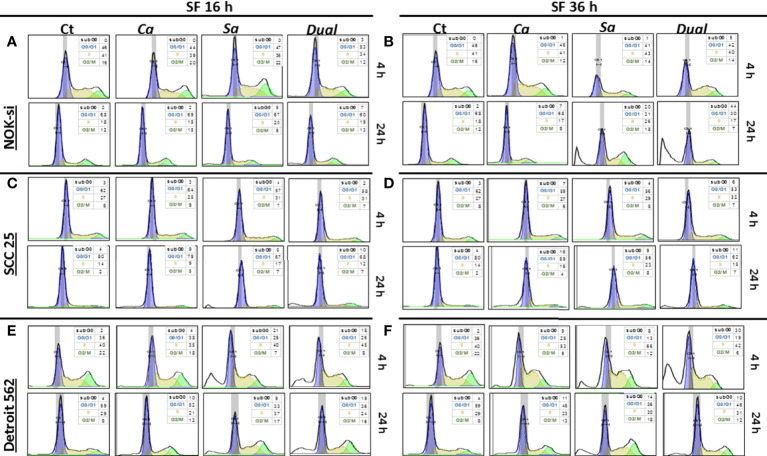
Cell cycle distribution histogram. The distribution rate of the cell cycle populations G0/G1 (blue), S (yellow), and G2/M (green) were analyzed for cell lines NOK-si **(A, B)**, SCC 25 **(C, D)**, and Detroit 562 **(E, F)** upon 4 h and 24 h of stimulation with soluble factors (SF) of C. albicans (Ca), S. aureus (Sa), and Dual species (D) from early (16-h) **(A, C, D)** and mature (36-h) **(B, D, F)** biofilms. The box inside of each histogram brings the distribution rate (%) of each cell cycle population. Sub G0/G1: population seen to the left of the G0/G1 peak. The Watson Pragmatic model was performed using the FlowJo ™ Software v10 program.

The cell cycle distribution was analyzed after 4 and 24 h of stimulation with SF from Ca, Sa, and D of 16-h and 36-h biofilms to verify some alterations in the proliferation of cell lines NOK-si, SCC 25, and Detroit 562 (**Figure 2**). A significant change was not observed in the cell cycle distribution of cell line NOK-si stimulated with SF of 16-h biofilm (**Figure 2A**) (both times performed) when compared with control Ct without a stimulus. However, higher levels of the sub-G0 population were observed when these cells were stimulated with SF from 36-h biofilm (**Figure 2B**) for 24 h, mainly for SF from D biofilm. A significant increase in the G2/M population was observed for the cells stimulated with SF from Sa. A slight reduction in the G1 population was showed after 24 h of stimulation with SF from Sa and D of 36-h biofilm (**Figures 3A, B**). SCC 25 cells (**Figures 2C, D**) showed a higher level of the sub-G0 population, mainly after 24 h of stimulation with SF from Ca, Sa, and D of 16-h biofilm. Twenty-four hours of stimulation with SF from Sa and D of 16-h and 36-h biofilms also promoted a reduction in the G1 population (**Figures 3D, E**). On the other hand, for the Detroit 562 cell line (**Figures 2E, F**), a higher level of the sub-G0 population was observed after 4 h of stimulation; SF from Sa and D of 16-h and 36-h biofilms after 4 h and 24 h of stimulation produced a reduction in the G1 population (**Figures 3F, G**). Similar results were observed when these cells were stimulated for 24 h with SF from Ca of 36-h biofilm. For this cell line, a significant tendency to increase the level of the G2/M population after 24 h of stimulation was also observed.

### Proto Oncogenes and Cell Cycle Gene Expression

**Figure 4:**
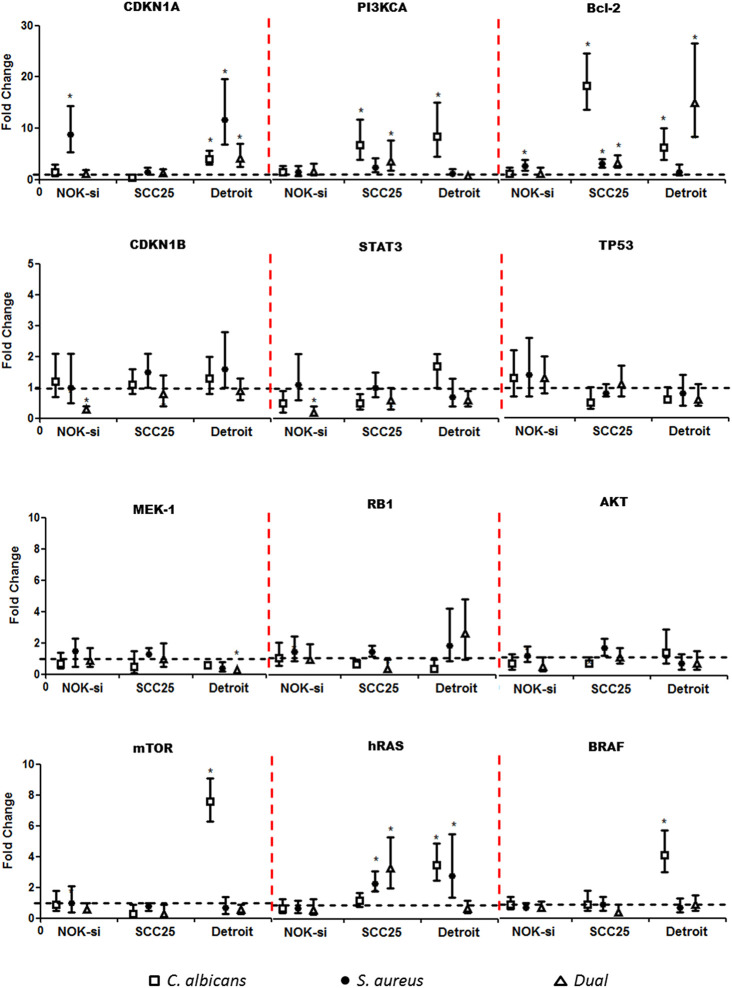
Proto-oncogenes and cell cycle gene expression upon SF from early biofilms. Gene expression was evaluated using the RT-qPCR analysis of NOK-si, SCC 25, and Detroit 562 upon 4 h of stimulation with soluble factors (SF) of *C. albicans* (Ca) (□), *S. aureus* (Sa) (•), and dual species (D) (Δ) from early 16-h biofilms. Data are shown as fold change values compared with cells stimulated with RPMI-1640 medium (control – black line) supplemented using the 2^-ΔΔCt^ method (*HPRT* was used as the internal control). *IC 95% means a significant difference vs. the control.

**Figure 5:**
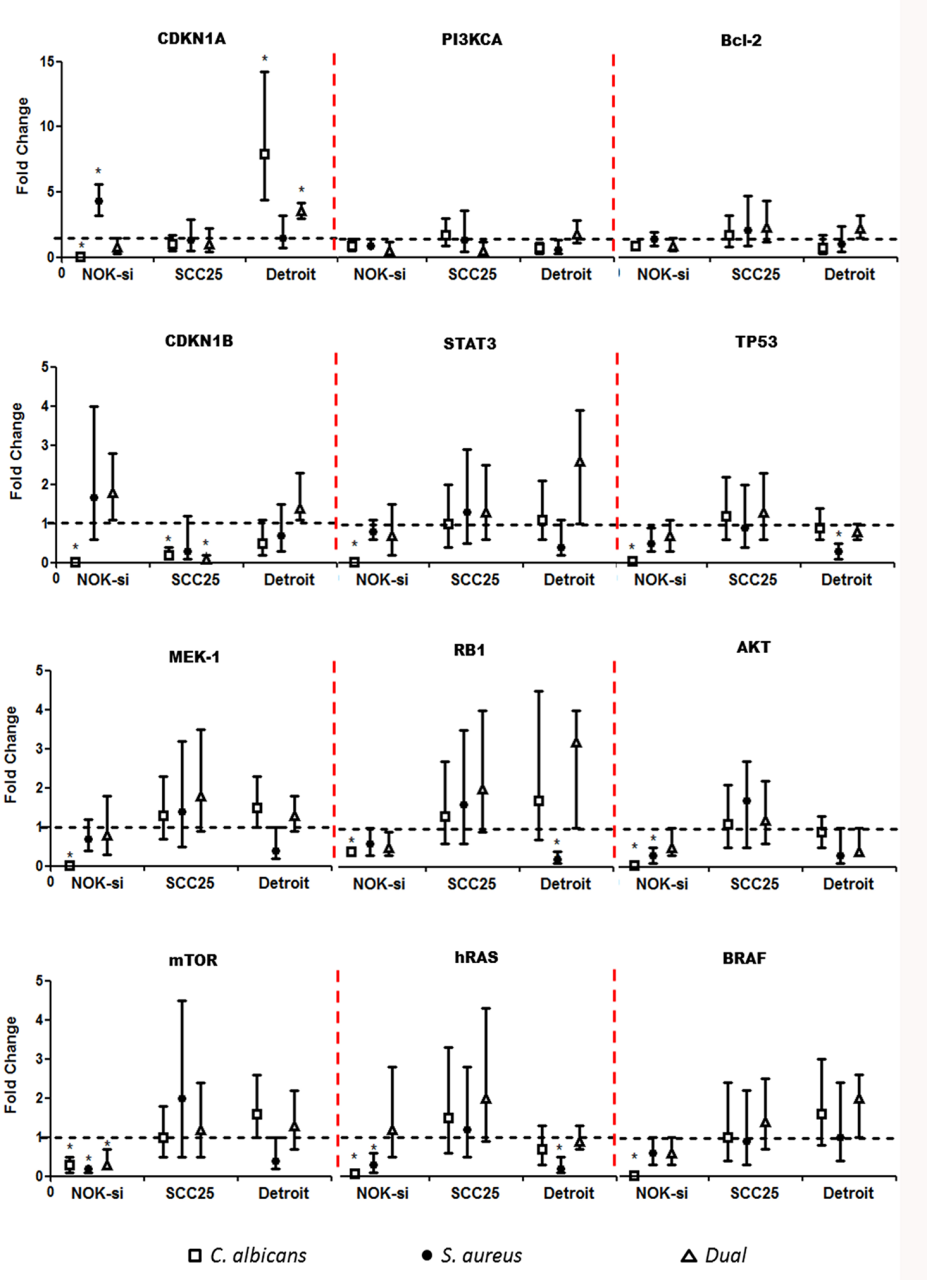
Proto-oncogenes and cell cycle gene expression upon SF from mature biofilms. Gene expression was evaluated by the RT-qPCR analysis of NOK-si, SCC 25, and Detroit 562 upon 4 h of stimulation with soluble factors (SF) of *C. albicans* (Ca) (□), *S. aureus* (Sa) (•), and dual species (D) (Δ) from mature 36-h biofilms. Data are shown as fold change values compared with cells stimulated with RPMI-1640 medium (control – black line) supplemented using the 2^−ΔΔCt^ method (*HPRT* was used as internal control). *IC 95% means a significant difference vs. the control.

Quantitative RT-PCR was performed after 4 h of stimulation with SF from Ca, Sa, and D of 16-h and 36-h biofilms to verify if there were alterations in the gene expression of hRAS/MEK and PI3KCA/AKT/*mTOR* signaling pathways on NOK-si, SCC 25, and Detroit 562 cells (**Figures 4** and **5**). For NOK-si, SF from Sa (16-h and 36-h biofilms) induced the overexpression of *Bcl-2* and *CDKN1A* genes (**Figures 4** and **5**). The 36-h SF from Ca promoted a significant decrease in *hRAS*, *BRAF*, *MEK1*, *AKT*, and *mTOR* gene expression, as well as *TP53*, *CDKN1A*, and *CDKN1B* cell cycle genes (**Figure 5**). Furthermore, SF came from D downregulated *CDKN1B*, *STAT3*, and *mTOR* genes. This downregulation was also observed after stimulation with SF from Sa of 36-h biofilm in *AKT*, *mTOR*, and *hRAS* genes (**Figures 4** and **5**). Both of the tumor cells stimulated with SF from biofilms presented changes in the relative levels of expression. SF from Ca, Sa, and D of 16-h biofilm promoted the overexpression of the *Bcl-2* antiapoptotic gene in the SCC 25 cell line (**Figure 4**). Oncogene *PI3KCA* was overexpressed upon stimulation with SF from Ca and D, and the overexpression of the *hRAS* gene was found after stimulation with SF from Sa and D (**Figure 4**). The SF from Ca of 36-h biofilm promoted the downregulation of the *CDKN1B* gene. After stimulation with SF from Ca, Sa, and D of 16-h biofilm, Detroit 562 cells showed the overexpression of the *CDKN1A* gene. Similar results were also seen when these cells were stimulated with SF from Ca and D of 36-h biofilm (**Figures 4** and **5**). Stimulation with SF from Ca of 16-h biofilm produced the overexpression of *hRAS*, *BRAF*, *PI3KCA*, and *mTOR* oncogenes, as well as antiapoptotic gene *Bcl-2*. Furthermore, SF from D of 16-h biofilm produced the overexpression of *Bcl-2* and the downregulation of *MEK1* genes (**Figure 4**). The SF from Sa of 36-h biofilm stimulated the downregulation of *TP53*, *RB1*, and *hRAS* genes (**Figure 5**).

## Discussion

The oral microbiota plays an important role in the human microbiome and in human health. A dysbiotic relationship exists between microbes and host results in the development of inflammatory diseases and cancer (Zhang et al., 2020). Oral pathogens produce toxic metabolites and toxins that can damage host DNA. The circulation of toxic metabolites may contribute to cancer onset or progression at locations distant from where a particular microbe resides (Rajagopala et al., 2017). Recent advances in the study of virulence factors from oral microbiota have suggested that these metabolites could stimulate head and neck carcinogenesis via different mechanisms, such as chronic inflammation, the inhibition of cellular apoptosis, the activation of cell proliferation, and the production of carcinogenic substances (Perera et al., 2016; Karpinski, 2019). This study evaluated the modulation in the gene expression of proto-oncogenes, the cell cycle profile, and cell viability in the oral epithelial cells (normal and neoplastic) after stimulation for 4 h or 24 h with SF of *C. albicans* (Ca), *S. aureus* (Sa), and dual species (D) of early (16-h) and mature (36-h) biofilms.

The tumor microenvironment is heterogeneous, composed of inflammatory tumor-infiltrating cells, tumor-associated fibroblasts, endothelial progenitor cells, different phenotypes of tumor cells, and inflammatory mediators (Tomasetti et al., 2017). The oral microbiome is complex, with more than 700 species in the oral cavity. The complex network influences the biological behavior of the tumor. However, in this study, we aimed to analyze proto-oncogenes and cell cycle gene expression promotes by high prevalence microorganisms in three different immortalized cell lines, that are not clinical samples but provides a better way to understand tumor target genes and the influence of two specific microorganisms found in oral biofilms. Besides that, the tumor cell lines SCC25 and Detroit 562 represent genetic changes encountered in EGFR/RAS/RAF/MEK/ERK and EGFR/PI3K/AKT/mTOR signaling (Supplementary materials) that play a pivotal role in cancer development and resistance (Ancrile et al., 2007).

SF from mature (36-h) Ca, Sa, and D of biofilms have demonstrated the ability to promote an inflammatory response and cell death in the normal oral epithelial cells of NOK-si (de Carvalho Dias et al., 2017). Based on these findings, we started to evaluate the SF’ role in oral epithelial cells (normal and neoplastic). The oral cell line with the normal phenotype of NOK-si used in this study showed overexpression in the *Bcl-2* gene after exposure to SF from Sa of 16-h biofilm. *Bcl-2* is an antiapoptotic activity gene, whose function is related to cell protection against cytotoxic stimuli (Ramírez-Garcia et al., 2014).

This fact could be correlated with the viability and cell cycle data, as the significant reduction of the viability index was not observed, and an increase in the sub-G0 population (apoptotic cells) also was not observed. The overexpression of *CDKN1A*, a cyclin-dependent kinase inhibitor associated with cell cycle G1 progression, and *Bcl-2* genes followed by the maintenance of cell viability could play an important role in cell survival upon stress conditions (Wilson et. al., 2001). In addition, *Bcl-2* overexpression is related to changes in the apoptosis pathway and in the subsequent progression of the tumor (Solomon et al., 2010). When the profile expression of these genes was analyzed upon 4 h of stimulation with SF of 36-h biofilms, changes in *Bcl-2* gene expression were not observed. However, *CDKN1A* was also found to be upregulated after 36-h Sa stimulation. In this condition, cell viability was not reduced, and the cell cycle profile after 24 h of stimulation increased the G2/M population. These results point to the fact that the upregulation of the *CDKN1A* gene is a major genetic event in the aberrant cell signaling survival that Sa SF from early and mature biofilms promote.

On the other hand, when we observed the gene expression profile of NOK-si upon stimulation with SF from Ca of 36-h biofilms, we observed a significantly decreased expression of *mTOR*, *STAT3*, *hRAS*, *BRAF*, *MEK1*, and *AKT* oncogenes, as well as *TP53*, *CDKN1A*, and *CDKN1B* cell cycle genes. *mTOR* and *STAT3* genes are recognized as regulators of a variety of critical functions, including cell survival, metabolism, cell growth, and proliferation (Zhou et al., 2018; Tan et al., 2019). The decrease in the expression level of these genes may explain the significant reduction in the cell viability index of NOK-si, mainly after 24 h of stimulation. Similar results were observed after stimulation with SF from D of 16-h and 36-h biofilms, with a reduction in the gene expression of *CDKN1B*, *STAT3*, and *mTOR*, suggesting that the normal phenotype cells of NOK-si are more susceptible to cell death signaling by *C. albicans* metabolites than by neoplastic cells SCC 25 and Detroit 562.

As observed for NOK-si, the SCC 25 cell line showed the overexpression of the *Bcl-2* gene after the cells were stimulated with SF from 16-h Sa biofilm. Such a result is probably related to the cell protection mechanism against a cytotoxic stimulus. In SCC 25 cells, with this stimulus, the significant superexpression of the *hRAS* gene was also observed, which has been related to the MAP kinase pathway and is responsible for promoting cell survival (Chang et al., 2014). These data could be corroborated with the increase in the viability index after 4 h. The overexpression of the *Bcl-2* gene was also observed in the SCC 25 cell line after stimulation with SF from Ca of 16-h biofilm. However, no significant reduction in cell viability was recorded, even after 24 h of stimulation. These results may indicate that in this neoplastic phenotype, the upregulation of *Bcl-2* may be a major event for cell survival, versus *CDKN1A*. In addition, this cell line displayed the overexpression of the *PI3KCA* gene, which promotes cell growth, the evasion of apoptosis, and tumor invasion when overexpressed in neoplastic cells. This contributes to tumor formation and progression (García-Escudero et al., 2018). Interestingly, no changes in the cell cycle profile were observed. The overexpression of *Bcl-2*, *PI3KCA*, and *hRAS* genes, which are also involved in promoting cell survival (Chang et al., 2014), was also noted after stimulation with SF from D of 16-h biofilm. SCC 25 cells stimulated with SF from Ca and D of 36-h biofilm showed the downregulation of the *CDKN1B* gene. This gene is a cyclin-dependent kinase inhibitor, which plays a significant role in the negative regulation of the cell cycle, mainly during G0 and G1 phases, and it is required for cellular transition from quiescence to a proliferative state (Hashmi et al., 2019). Clinically, *CDKN1B* downregulation is correlated with poor prognosis and the more aggressive tumor phenotype of HNSCC (Hashmi et al., 2019).

For the Detroit 562 cell line, upon stimulation with SF from Sa of 16-h biofilm, the overexpression of the *CDKN1A* gene was observed, and so was cell survival, even after 24 h of stimulation. The increased expression of the *CDKN1A* gene in HNSCC has been correlated with nodal metastases, tumor recurrence, and decreased survival (Sadaf et al., 2015). The possible mechanism of action with HNSCC is related to its anti-apoptotic effect and its uncontrolled cell proliferation (Sadaf et al., 2015) as shown by our results revealing a significant tendency to increase the G2/M phase.

Similar outcomes were observed after stimulation with SF from Ca and D of 16-h and 36-h biofilms. Thus, these biofilms could act as mitogens in Detroit 562 cells. SF from Ca of 16-h biofilm lead to the overexpression of *hRAS* and *BRAF* genes. These are related to the MAP kinase pathway, which is responsible for promoting cell survival (Chang et al., 2014). These facts suggest that *C. albicans*, with or without *S. aureus*, produces metabolites that may stimulate the survival of metastatic cells in advanced tumor lesions. The same stimulus promoted the overexpression of the *PI3KCA* gene, which has been correlated with the advanced HNSCC stage, lymph node metastasis, and the development of resistance to chemotherapy (Fenic et al., 2007; Jung et al., 2018). Thus, SF from Ca of 16-h biofilm could promote changes in the tumor environment, leading to different responses in metastatic (survival) and normal cells (death). SF from Ca and D of 16-h biofilm also promoted the overexpression of the *Bcl-2* gene. The expression of the *Bcl-2* gene in the context of the clinical-pathological characteristics of HNSCC has shown that a positive correlation exists between the overexpression of this gene in tumor cells and the tumor mitotic index, a higher index of atypical mitoses, and a micro focal pattern of the invasive margin of the tumor, thus promoting unfavorable histopathological characteristics (Sulkowska et al., 2003). SF from Sa of 36-h biofilm induced *TP53* and *RB1* downregulation, maintained Detroit 562, and increased the G2/M phase, even after 24 h of stimulation.

## Conclusion

SF from single- and dual-species biofilms of *C. albicans* and *S. aureus* promoted changes in proto-oncogenes and cell cycle gene expression in normal and neoplastic oral epithelial cell lines. SF from both early and mature *S. aureus* biofilms induced the overexpression of *Bcl-2* and the *CDKN1A* gene in the normal and neoplastic cell lines evaluated in this study, and they enhanced cell survival and the G2/M cell population. *C. albicans* and SF from 16-h and 36-h biofilms caused disturbances in cell viability and in the cell cycle profile, including the downregulation of genes in terms of cell growth and survival in normal oral epithelial cells. However, the same stimulus in neoplastic cell lines modulated the overexpression of *hRAS*. *PI3KCA*, and *BRAF* oncogenes with a cell survival profile. In summary, this study contributed to elucidating how biofilm metabolites could modulate gene expression and tumor cell survival in a cell phenotype–dependent manner.

## Data Availability Statement

The raw data supporting the conclusions of this article will be made available by the authors, without undue reservation.

## Author Contributions

CV contributed to conception, design of the study, and financial support. PB contributed to conception, design of the study, and all analysis, interpretation of results, and elaboration of the manuscript. MA performed all assays and contributed with elaboration of the manuscript. AS contributed with cell cycle assay by flow cytometer, analysis and interpretation of results, and elaboration of the manuscript. GN and VV helped to perform whole molecular assays. All authors contributed to the article and approved the submitted version.

## Funding

This research study received support from the Brazilian governmental agencies of Fundação de Amparo à Pesquisa do Estado de São Paulo (FAPESP, # 15/50311-8), Conselho Nacional de Desenvolvimento Científico e Tecnológico (CNPq, # 304247/2017-0), Colombian governmental agencies Departamento Administrativo de Ciencia, Tecnología e Innovación (COLCIENCIAS, convocatória doctorados en el exterior 2015), and Agencia de Educación Superior de Medellín (SAPIENCIA, programa ENLAZAMUNDOS 2019-2).

## Conflict of Interest

The authors declare that the research was conducted in the absence of any commercial or financial relationships that could be construed as a potential conflict of interest.
